# Normal Ranges of Right Atrial Strain and Strain Rate by Two-Dimensional Speckle-Tracking Echocardiography: A Systematic Review and Meta-Analysis

**DOI:** 10.3389/fcvm.2021.771647

**Published:** 2021-12-17

**Authors:** Ali Hosseinsabet, Roshanak Mahmoudian, Arash Jalali, Reza Mohseni-Badalabadi, Tahereh Davarpasand

**Affiliations:** ^1^Department of Cardiology, Tehran Heart Center, Tehran University of Medical Sciences, Tehran, Iran; ^2^Department of Research, Tehran Heart Center, Tehran University of Medical Sciences, Tehran, Iran

**Keywords:** right atrium, speckle-tracking echocardiography, strain, normal range, meta-analysis

## Abstract

**Background:** Normal range values of right atrial (RA) phasic function markers are essential for the identification of normal and abnormal values, comparison with reference values, and the clinical meaning of obtained values. Accordingly, we aimed to define the normal range values of RA phasic function markers obtained by 2D speckle-tracking echocardiography through a meta-analysis and determine the main sources of heterogeneity among reported values.

**Methods:** PUBMED, SCOPUS, and EMBASE databases were searched for the following keywords: “right atrial/right atrium” and “strain/speckle/deformation” and “echocardiography.” Studies were selected that included a human healthy adult group without any cardiovascular diseases or risk factors and that were written in the English language. For the calculation of each marker of RA phasic functions, a random-effect model was used. Meta-regression was employed to define the major sources of variabilities among reported values.

**Results:** Fifteen studies that included 2,469 healthy subjects were selected for analysis. The normal range values for RA strain and strain rate were 42.7% (95% CI, 39.4 to 45.9%) and 2.1 s^−1^ (95% CI, 2.0 to 2.1 s^−1^) during the reservoir phase, respectively, 23.6% (95% CI, 20.7 to 26.6%) and −1.9 s^−1^ (95% CI, −2.2 to −1.7 s^−1^) during the conduit phase, correspondingly, and 16.1% (95% CI, 13.6 to 18.6%) and −1.8 s^−1^ (95% CI, −2.0 to −1.5 s^−1^) during the contraction phase, respectively. The sources of heterogeneity for the normal range of these markers were the number of participants, the type of software, the method of global value calculation, the right ventricular fractional area change, the left ventricular (LV) ejection fraction, the RA volume index, sex, the heart rate, the diastolic blood pressure, the body mass index, and the body surface area.

**Conclusions:** Using 2D speckle-tracking echocardiography, we defined normal values for RA phasic function markers and identified the sources of heterogeneity as demographic, anthropometric, hemodynamic, and echocardiography factors.

**Systematic Review Registration:** https://www.crd.york.ac.uk/prospero/display_record.php?ID=CRD42021236578, identifier: CRD42021236578.

## Introduction

The right atrium (RA) is pivotal for blood entrance to the heart. It manages not only right ventricular (RV) filling during diastole by reserving the blood during systole but also the delivery of the stored blood during early diastole and further RV filling by contraction in late diastole. Unlike the left atrium (LA), the RA interacts with a lower pressure chamber, the RV, which has less myocardial mass than the LV (1).

RA phasic functions can be evaluated by several methods such as echocardiography and cardiac magnetic resonance (2, 3). Nonetheless, echocardiography has been the main method for the assessment of RA phasic functions because of its availability and low cost. Consequently, the recent decades have witnessed the advent of several echocardiographic modalities for the evaluation of RA phasic functions (4, 5). Among these modalities, 2D speckle-tracking echocardiography (2DSTE) is prominent because of its angle independence when compared with tissue Doppler imaging, low load dependency when compared with volumetric methods in normal subjects, and relative resistance against translational motion (6–8). The 2DSTE modality can evaluate RA phasic functions in healthy subjects and demonstrate impairment in various disorders such as diabetes and pulmonary hypertension (4, 9–23). This echocardiographic modality also has a prognostic role in pulmonary hypertension and post-myocardial infarction events (24–26). Comparison between feature-tracking magnetic resonance imaging and 2DSTE demonstrates a good agreement between these two methods in the assessment of the deformation parameters of RA phasic functions (27). Although there is a consensus regarding how to measure the deformation indices of RA phasic functions by 2DSTE (28), the lack of a normal reference range for comparison impedes more clinical usage of this method.

In this study, we drew upon a systematic review and a meta-analysis to obtain the normal ranges of various 2DSTE-derived markers of RA phasic functions and to clarify the main sources of heterogeneity in their reported values.

## Methods

### Search Profile

On April 29, 2021, we searched PUBMED, SCOPUS, and EMBASE databases *via* the following keywords: “right atrial/right atrium” and “strain/speckle/deformation” and “echocardiography”. The search was limited to studies in the English language (Supplementary Material 1). References were also searched to find other related studies. We applied the Preferred Reporting Items for Systematic Reviews and Meta-Analyses (PRISMA) guidelines (29). On March 13, 2021, our study was recorded in the Prospero database (CRD42021236578).

### Study Selection

The inclusion criteria were composed of the evaluation of the RA by 2DSTE or velocity vector imaging and the inclusion of a normal healthy control group without any cardiovascular diseases or risk factors. The exclusion criteria consisted of animal studies, conference articles, case reports, editorials, letters to the editor, review articles, articles without abstracts, the inclusion of subjects below 18 years of age, and RA evaluation by tissue Doppler imaging. Also excluded were studies that used the same data set. (The exception was one article featuring a large study population). If articles had the same number of subjects, the article citing the gating for measurements was selected. Additionally, studies that presented the values separately for sex subgroups were excluded. The titles and abstracts of the studies selected from the aforementioned databases were reviewed by three independent researchers (R.M., R.M.B., and A.H.). Discordances among the reviewers were resolved by discussion between A.H. and T.D.

### Data Collection

R.M., R.M.B., and A.H. independently reviewed the full text of the eligible studies. The demographic characteristics, clinical information, and echocardiography data (including the deformation markers of RA phasic functions) of the control group were recorded. Discordances between the three aforementioned researchers were resolved through discussion between A.H. and T.D. The studies that seemed to have used the same or overlap data sets were excluded. (The exception was one article, the control group of which had the highest number of subjects of all the studies).

### Statistical Analysis

Stata software, Release 16 (College Station, TX: StataCorp LLC), was used for statistical analysis. A random-effects model was employed to calculate the mean and the 95% confidence interval (CI) for each phasic strain and strain rate. Heterogeneity and inconsistency among the selected studies were assessed by Cochrane's Q test (*P* < 0.1) and I2 statistic, respectively. The results pertaining to each phasic strain and strain rate were demonstrated as forest plots. Reported demographic characteristics, clinical findings, and echocardiography data were considered sources of heterogeneity concerning each phasic strain and strain rate, and the effects of these variables on the variation of the normal range of each phasic strain and strain rate were assessed by meta-regression. Through a comparison between the results of the random-effects model and a fixed-effects model, the stability of the estimated normal range for each phasic strain and strain rate was checked. Egger's test (*P* < 0.1) and funnel plots were utilized to evaluate publication bias.

The criteria recommended by Downs and Black for the evaluation of the quality (internal and external validity) of studies were drawn upon (30). Well-defined methods were used in the reporting of inter and intraobserver variabilities, the heart rate, systolic and diastolic blood pressures, the phasic strain, and the phasic strain rate. The blindness of the operator who obtained images and the echocardiographer who analyzed videos was considered an additional criterion for quality in keeping with previously published systematic reviews and meta-analyses in this context. R.M., R.M.B., and A.H. independently checked the quality of the studies selected, and the differences in their assessments were resolved through an agreement between A.H. and T.D.

## Results

### Study Selection

**Figure 1:**
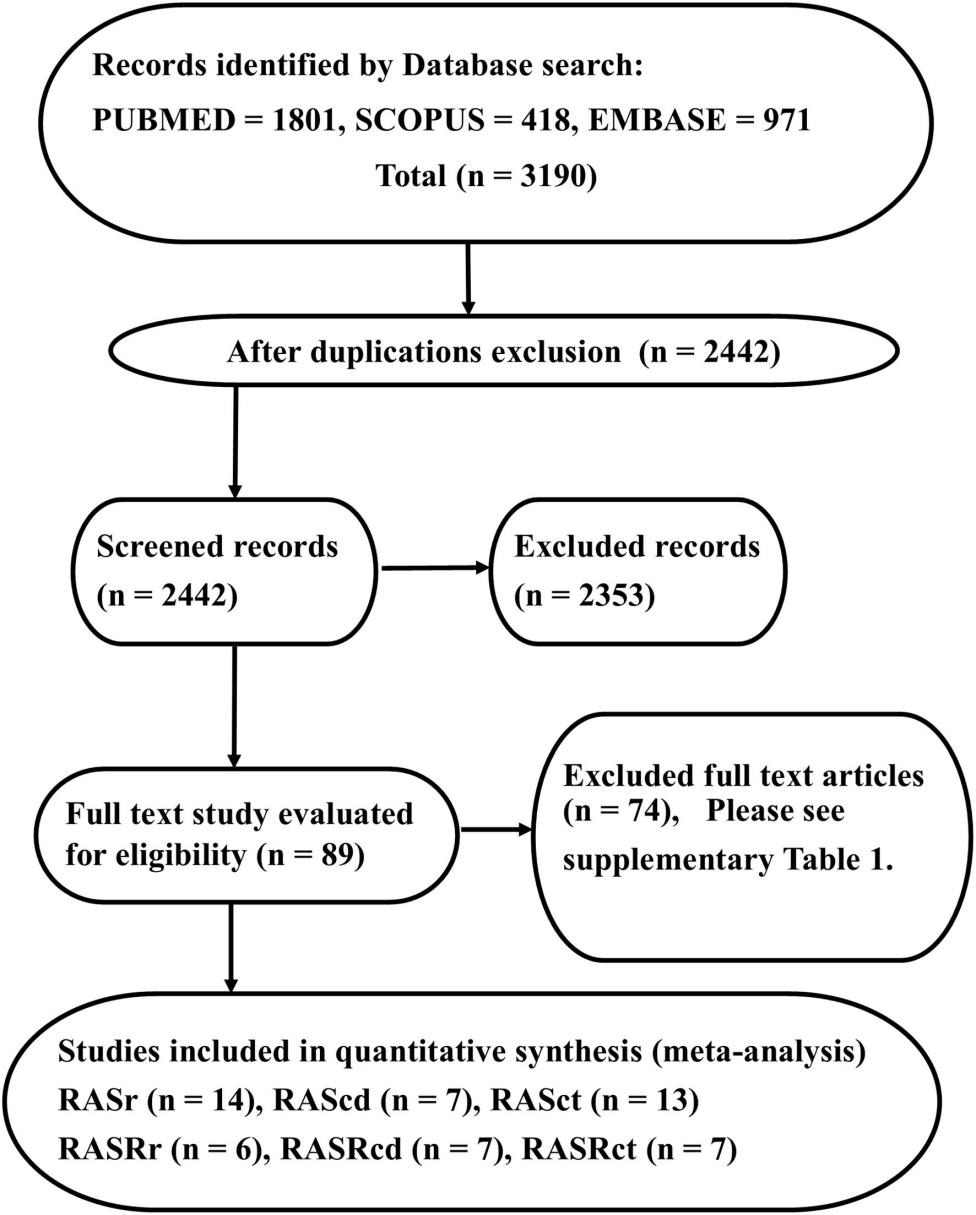
The image presents the study design and the Preferred Reporting Items for Systematic Reviews and Meta-Analyses flowchart, illustrating the selection process of studies. The reasons for full-text exclusion are demonstrated in Supplementary Table 1.

The PRISMA diagram of our study is presented in Figure 1. Our database search yielded 3,190 studies. Following the exclusion of duplicate studies, 2,442 studies were selected for title and abstract review. Eighty-nine studies were identified as suitable for full-text review. Our reference search failed to identify any other studies.

Next, 74 studies were excluded (Supplementary Table 1), and 15 studies were considered eligible for further analysis. Fourteen studies (2,469 subjects) featured RA strain assessment during the reservoir phase (RASr), seven studies (2,112 subjects) assessed RA strain during the conduit phase (RAScd), 13 studies (2,409 subjects) offered RA strain assessment during the contraction phase (RASct), six studies (269 subjects) evaluated the peak RA strain rate during the reservoir phase (pRASRr) and the peak RA strain rate during the conduit phase (pRASRcd), and seven studies (299 subjects) presented peak RA strain rate assessment during the contraction phase (pRASRct) (Table 1). The mean age of the participants in these studies ranged between 25 and 51 years, and male subjects comprised a range from 0 to 100%.

**Table 1:** Study characteristics.

**Study**	**Year**	**N**	**Age (y)**	**Male (%)**	**HR (bpm)**	**BMI (kg/m^**2**^)**	**BSA (m^**2**^)**	**SBP (mmHg)**	**DBP (mmHg)**	**LVEF (%)**	**RAVI (2D) (ml/m^**2**^)**	**RV FAC (%)**	**TAPSE (mm)**	**RVSm (cm/s)**	**SPAP mmHg**	**RV E/e^**′**^ ratio**	**FR (Hz)**	**Platform**	**Software**	**Probe**	**Gating**	**Model**	**Track ability (%)**	**Method of calculation**	**Measured deformation index**	**Disease studied**
Padeletti et al.	2012	103	34.1 ± 15.1	40.8	74.8 ± 14.6	22.4 ± 3.5	NR	118 ± 14.1	76.4 ± 9.1	61.87 ± 3.67	NR	NR	23 ± 3	14 ± 3	NR	NR	60–80	Vivid I,GE	EchoPac	NR	RR	6	93	Global	RASr, RASct, RASRr, RASRcd, RASRct	Healthy subjects for normal reference range
D'Ascenzi et al.	2013	78	25.20 ± 3.92	64	72.16 ± 13.11	NR	1.82 ± 0.18	NR	NR	NR	19.89 ± 4.99	NR	23.18 ± 3.27	14 ± 3	NR	4.11 ± 1.05	60–80	Vivid 7, GE	EchoPac	NR	RR	6	NR	Average	RASr, RASct	Athletics
Peluso et al.	2013	195	43 ± 15	44	68 ± 11	23 ± 3	1.78 ± 0.19	122 ± 14	74 ± 8	NR	23 ± 7	NR	NR	NR	NR	NR	73	Vivid E9,GE	EchoPAC v110.1.3	M5S	PP	NR	93	Global	RASr, RAScd, RASct	Healthy subjects for normal reference range
Pagourelias et al.	2013	26	26.6 ± 5.6	100	65 ± 9	22.4 ± 2.4	2.01 ± 0.16	120.6 ± 8	75 ± 9	NR	NR	42.7 ± 7.2	NR	14.8 ± 1.7	18.3 ± 8.6	4 ± 0.85	<90	Vivid S5, GE	EchoPAC	M3S	RR	6	NR	Average	RASr, RASct, RASRr, RASRcd, RASRct	Athletics
Gabrielli et al.	2014	20	27 ± 4	100	76 ± 12	23 ± 2.7	1.80 ± 0.12	NR	NR	60 ± 6	19.0 ± 5.1	40 ± 5	28 ± 3	NR	NR	NR	>50	Vivid Q,GE	EchoPac version 108.1.6	M4S	PP	6	NR	Average	RASr, RASct, RASRr, RASRcd, RASRct	Athletics
Durmus et al.	2015	40	45.9 ± 7.6	48	NR	NR	NR	NR	NR	64.5 ± 4.9	NR	33.8 ± 3.7	24.3 ± 3.4	12.9 ± 2.0	19.8 ± 6.2	NR	>40	Vivid 7,GE	EchoPAC 6.1	NR	RR	NR	NR	NR	RASr, RASct	Scleroderma
McClean et al.	2015	20	27 ± 8	100	63 ± 9	NR	1.96 ± 0.13	129 ± 18	81 ± 14	NR	23 ± 5	NR	NR	NR	NR	NR	40–90	Vivid Q,GE	EchoPAC 6.0	NR	RR	6	NR	Average	RASr, RAScd, RASct	Athletics
Querejeta Roca et al.	2015	30	49.2 ± 12.3	33	69 ± 11	23.9 ± 3.9	NR	124 ± 12	71 ± 9	61 ± 5	NR	54 ± 6	21.5 ± 1.1	14.2 ± 2.3	NR	NR	NR		Tomtec system	NR	NR	NR	NR	NR	RASr, RASRr, RASRcd, RASRct	Pulmonary arterial pressure
Tadic et al.	2015	60	51 ± 8	49	73 ± 7	24.4 ± 2.6	1.92 ± 0.15	121 ± 10	72 ± 8	64 ± 4	20.8 ± 4.6	NR	22 ±3	NR	21 ±4	4.5 ± 1.5	NR	Vivid 7,GE	EchoPAC 110.1.2	NR	RR	6	NR	Average	RASr, RAScd, RASct, RASRr, RASRcd, RASRct	Diabetes and prediabetes
Gabrielli et al.	2016	30	35 ± 4	100	69 ± 12	NR	1.9 ± 0.2	119 ± 6	77 ± 4	61 ± 5	22.6 ± 4.7	49 ± 7	NR	NR	NR	NR	>60	Vivid– Q; GE	EchoPac version 108.1.6	M4S	PP	6	97.5	Average	RASct, RASRct	Athletics
Brand et al.	2018	123	42.4 ± 10.9	0	71 ± 11	22.4 ± 2.8	NR	117 ± 14	71 ± 10	61 ± 5	NR	41.1 ± 10.5	24.4 ± 3.8	14.2 ± 1.9	20.0 ± 4.2	NR	NR	Vivid E9,GE	EchoPAC PC	M5S	RR	NR	92.7	Global	RASr, RAScd, RASct	Healthy subjects
Li et al.	2018	30	46.82 ± 5.45	57	71.55 ± 15.35	27.06 ± 4.38	NR	122.19 ± 6.69	73.51 ± 7.19	NR	24.9 ± 4.17	NR	NR	NR	24.31 ± 5.63	NR	70–90	Acuson SC2000 Ultrasound system; Siemens Medical Solutions	NR	NR	RR	NR	NR	Average	RASr, RASRr, RASRcd, RASRct	Sleep apnea
Can Bostan et al.	2020	70	33.9 ± 9.5	63	76 ± 11	NR	1.58 ± 0.30	123 ± 7	74 ± 7	54.6 ± 4.3	19.8 ± 6.8	41.3 ± 7.5	23.6 ± 3.2	14.6 ± 1.7	19.8 ± 8.0	NR	>50	Epiq 7, Philips	NR	S5-1	RR	6	NR	NR	RASr, RAScd, RASct	Smokers
Palmer et al.	2020	101	41 (30–52)	44	68 ± 11	25.27 ± 2.82	1.89 ± 0.21	123.38 ± 15.46	74.02 ± 11.21	62 ± 6	21 ± 6	52 ± 8	21 ± 4	NR	NR	NR	40– 80	EPIQ7, Philips	2D Cardiac Performance Analysis; TomTec Imaging Systems	X5-1	RR	3	NR	NR	RASr, RAScd, RASct	Healthy subjects
Soulat-Dufour et al.	2020	1543	47 ± 17	51	NR	NR	1.77 ± 0.22	120 ± 13	74 ± 9	NR	19.4 ± 6.0	NR	NR	NR	NR	NR	NR	NR	Image Arena; TOMTEC	NR	RR	NR	79	NR	RASr, RAScd, RASct	Healthy subjects

*BMI, Body mass index; BSA, Body surface area; DBP, Diastolic blood pressure; FR, Frame rate; HR, Heart rate; LVEF, Left ventricular ejection fraction; NR, Not reported; pRASRr, peak Right atrial strain rate during the reservoir phase; pRASRcd, peak Right atrial strain rate during the conduit phase; pRASRct, peak Right atrial strain rate during the contraction phase; RASr, Right atrial strain during the reservoir phase; RAScd, Right atrial strain during the conduit phase; RASct, Right atrial strain during the contraction phase; RAVI, Right atrial volume index; RV, Right ventricle; RVFAC, Right ventricular fractional area change; RVSm, Right ventricular systolic velocity; SBP, Systolic blood pressure; SPAP, Systolic pulmonary artery pressure; TAPSE, Tricuspid annular plane systolic excursion*.

### The Normal Ranges of RA Phasic Strain and Strain Rate

#### Reservoir Function Markers

**Figure 2:**
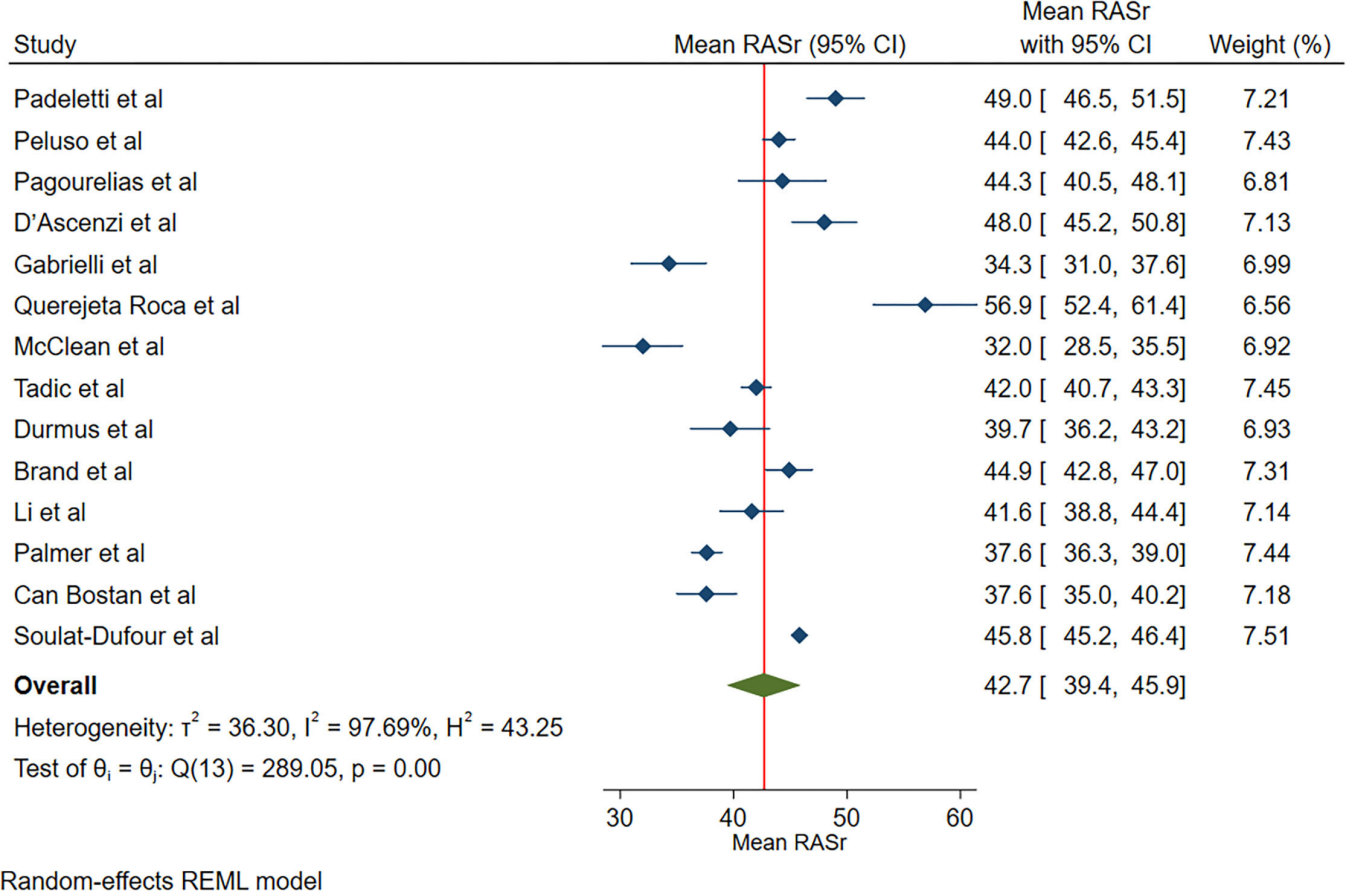
The image demonstrates the normal range of the longitudinal 2D speckle-tracking echocardiography-derived right atrial strain during the reservoir phase.

The reported mean normal value for RASr was 42.7% (95% CI, 39.4 to 45.9%), which ranged from 32.0 to 56.9%. Inter-study heterogeneity (Q = 289; *P* < 0.01) and inconsistency (I^2^ = 97.7%) were significant. The fixed-effects model demonstrated a mean RASr value of 43.7% (95% CI, 43.2 to 44.1%) (Figure 2).

**Figure 3:**
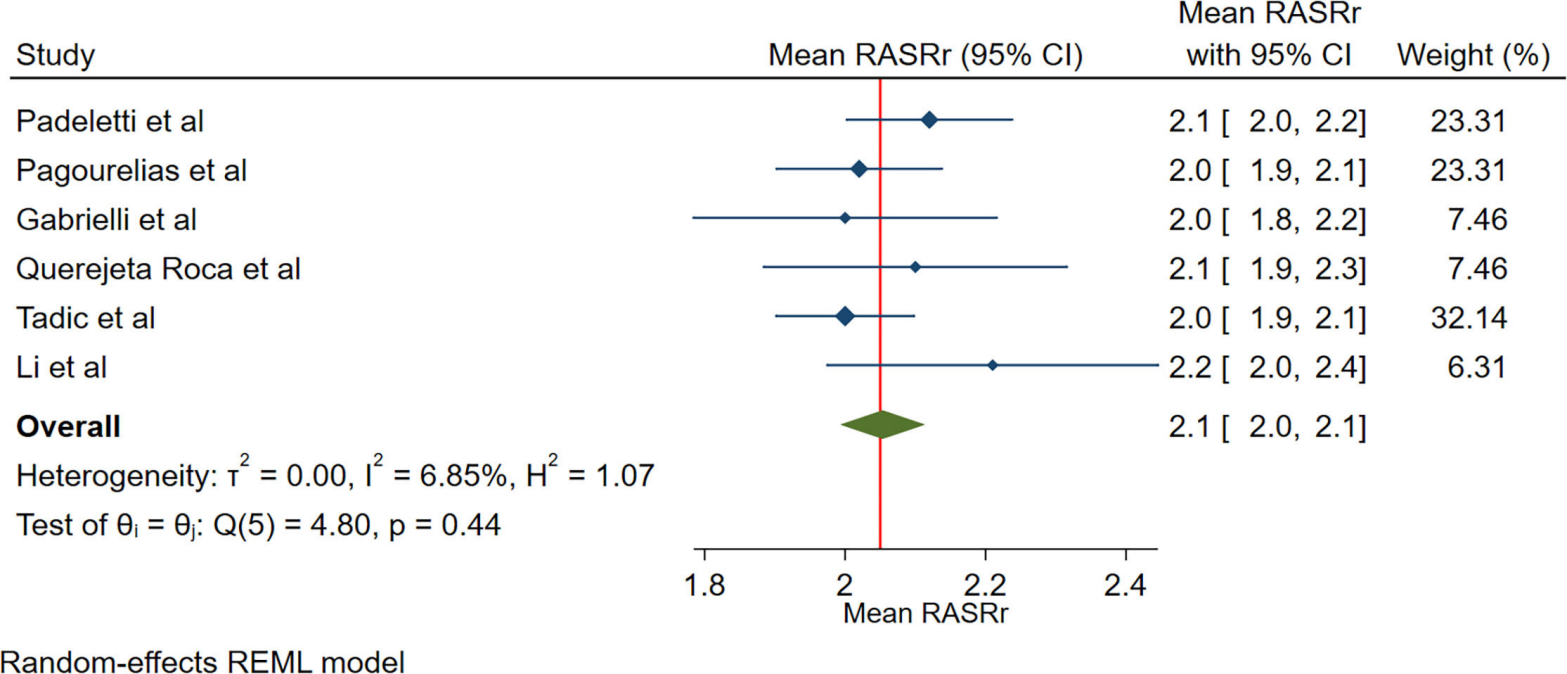
The image demonstrates the normal range of the longitudinal 2D speckle-tracking echocardiography-derived peak right atrial strain rate during the reservoir phase.

The reported mean normal value for pRASRr was 2.1 s^−1^ (95% CI, 2.0 to 2.1 s^−1^). The normal range of RASRr varied between 2.0 and 2.2 s^−1^. Inter-study heterogeneity (Q = 5; *P* = 0.44) and inconsistency (I^2^ = 6.9%) were non-significant. The fixed-effects model demonstrated a mean value pRASRr value of 2.1 s^−1^ (95% CI, 2.0 to 2.1 s^−1^) (Figure 3).

#### Conduit Function Markers

**Figure 4:**
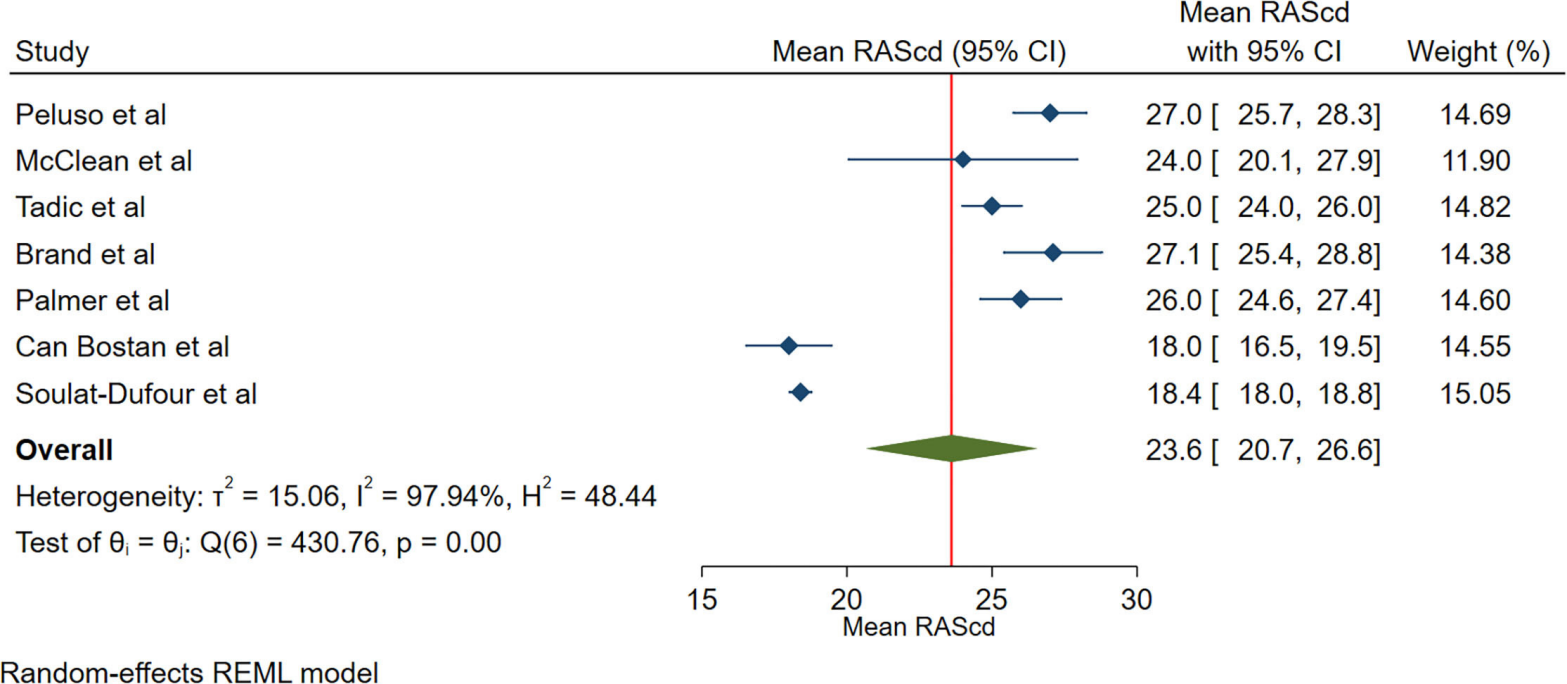
The image demonstrates the normal range of the longitudinal 2D speckle-tracking echocardiography-derived right atrial strain during the conduit phase.

The reported mean normal value for RAScd was 23.6% (95% CI, 20.7 to 26.6%). The normal range of RAScd varied between 18.0 and 27.1%. Inter-study heterogeneity (Q = 431; *P* < 0.01), and inconsistency (I^2^ = 98.0%) were significant. The fixed-effects model showed a mean RAScd value of 20.3% (95% CI, 19.9 to 20.6%) (Figure 4).

**Figure 5:**
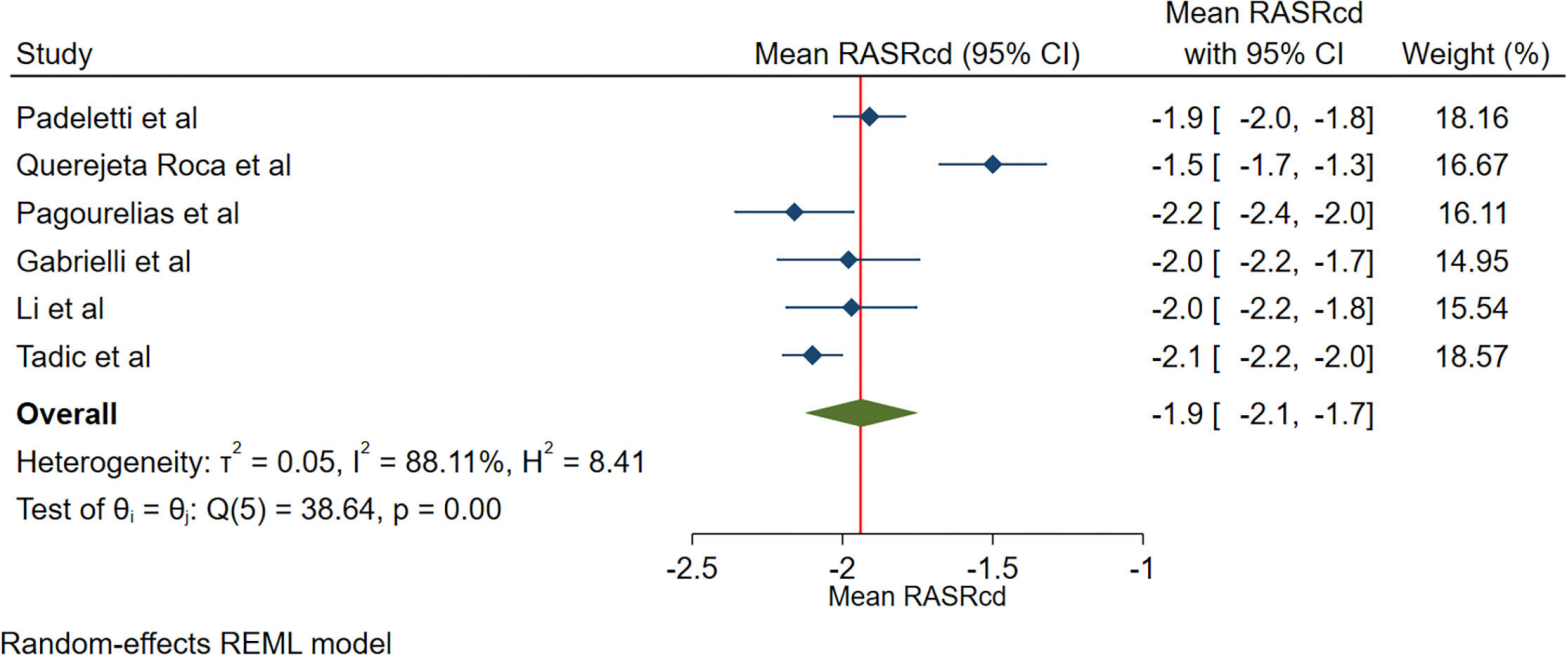
The image demonstrates the normal range of the longitudinal 2D speckle-tracking echocardiography-derived peak right atrial strain rate during the conduit phase.

The reported mean normal value for pRASRcd was −1.9 s^−1^ (95% CI, −2.2 to −1.7 s^−1^), which ranged from −2.2 to −1.5 s^−1^. Inter-study heterogeneity (Q = 37; *P* < 0.01) and inconsistency (I^2^ = 88.8%) were significant. The fixed-effects model demonstrated a mean pRASRcd value of −2.0 s^−1^ (95% CI, −2.1 to −1.9 s^−1^) (Figure 5).

#### Contraction Function Markers

**Figure 6:**
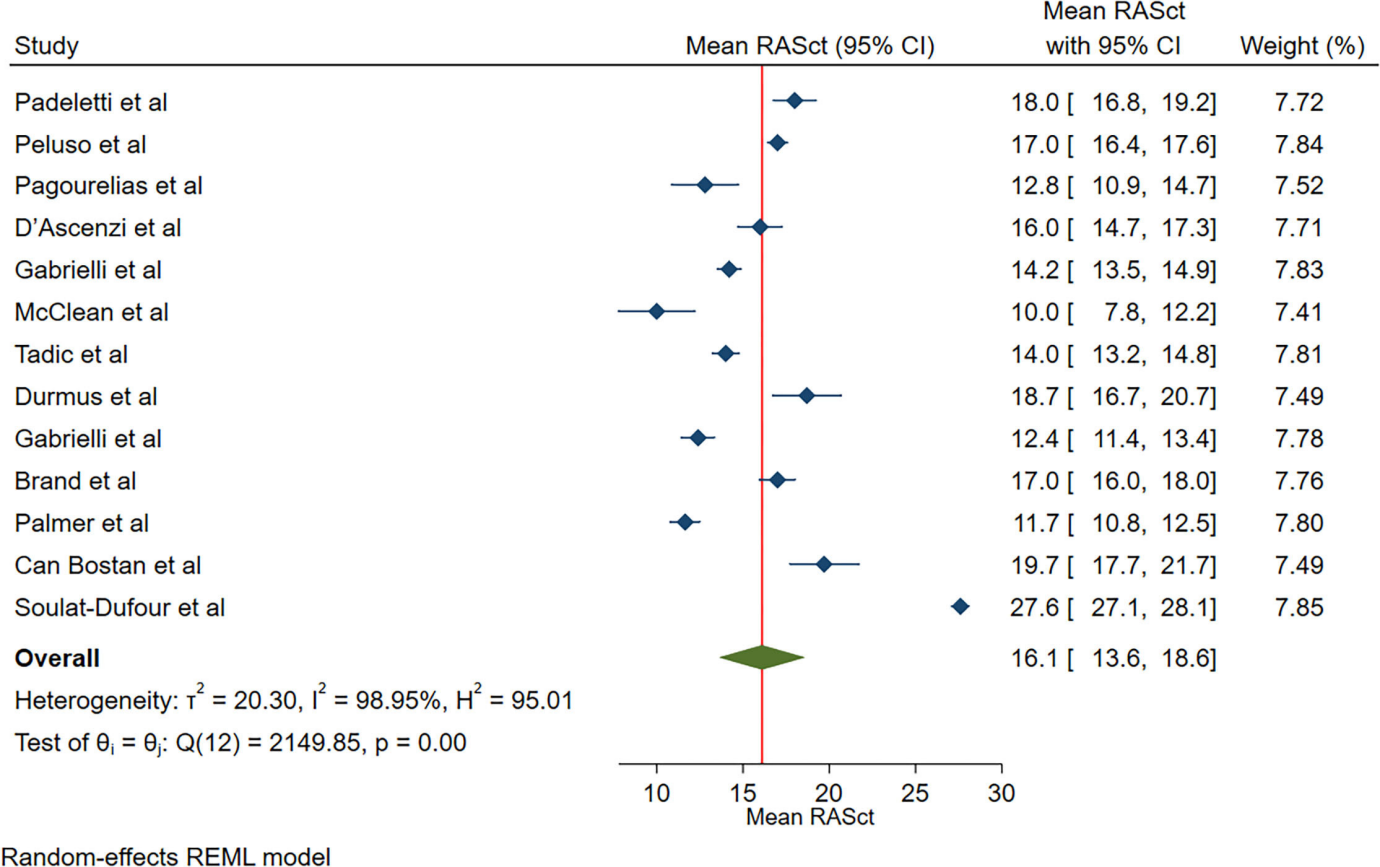
The image demonstrates the normal range of the longitudinal 2D speckle-tracking echocardiography-derived right atrial strain during the contraction phase.

The reported mean normal value for RASct was 16.1% (95% CI, 13.6 to 18.6%), which ranged from 11.7 to 27.6%. Inter-study heterogeneity (Q = 2,150; *P* < 0.01) and inconsistency (I^2^ = 99.0%) were significant. The fixed-effects model showed a mean RASct value of 18.1 % (95% CI, 17.9 to 18.4%) (Figure 6).

**Figure 7:**
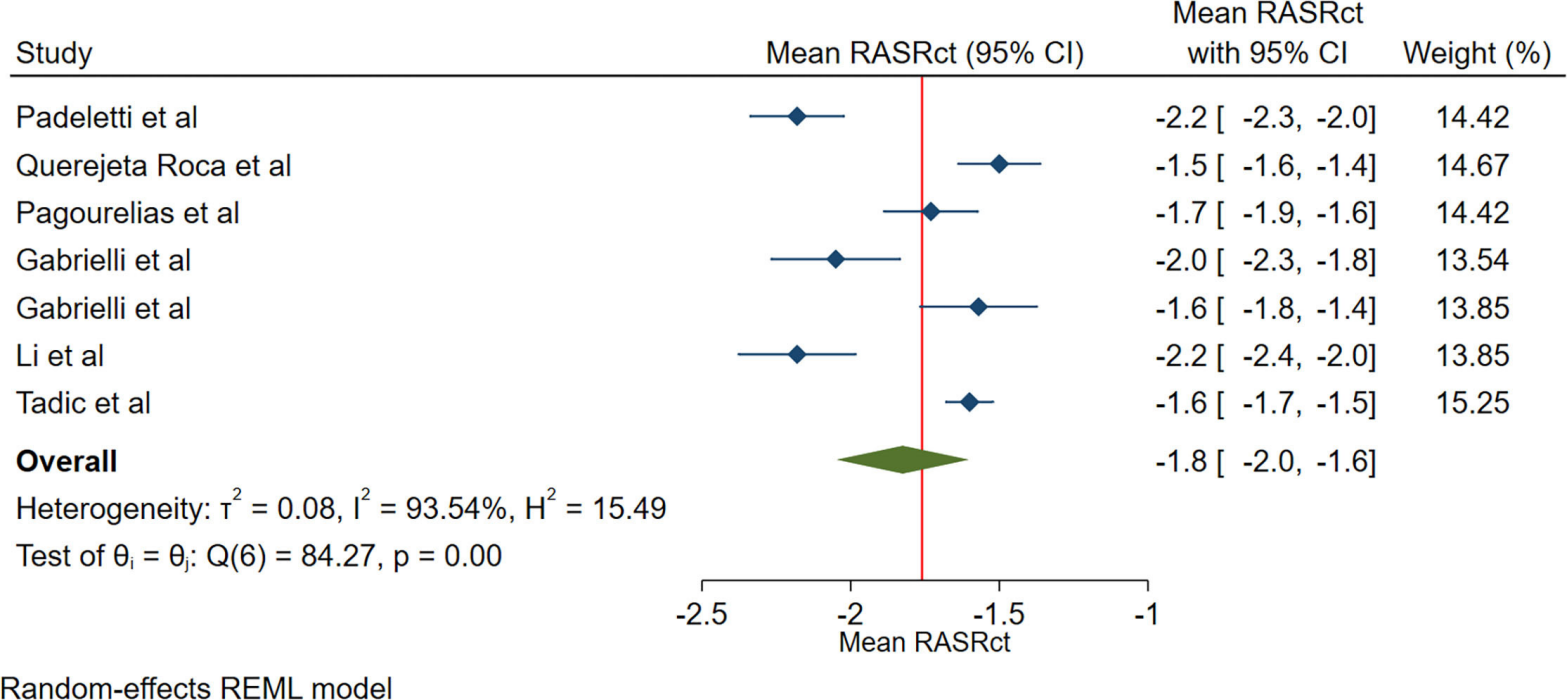
The image demonstrates the normal range of the longitudinal 2D speckle-tracking echocardiography-derived peak right atrial strain rate during the contraction phase.

The reported mean normal value for pRASRct was −1.8 s^−1^ (95% CI, −2.0 to −1.5 s^−1^). The normal range of pRASRct varied between −2.2 s^−1^ and −1.5 s^−1^. Inter-study heterogeneity (Q = 48; *P* < 0.01) and inconsistency (I^2^ = 92.3%) were significant. The fixed-effects model demonstrated a mean pRASRct value of −1.7 s^−1^ (95% CI, −1.7 to −1.6 s^−1^) (Figure 7).

### Meta-Regression

#### Reservoir Function Markers

In the case of RASr, sex (male) (β = −0.12; *P* = 0.028) and diastolic blood pressure (β = −1.13; *P* = 0.036) were sources of between-study heterogeneity. Apropos of pRASRs, between-study heterogeneity was not statistically significant (Table 2).

**Table 2:** Meta-regression analysis for longitudinal two-dimensional speckle tracking echocardiography derived right atrial strains and strain rates.

**Variables**	**RASr**			**RAScd**			**RASct**			**pRASRr**			**pRASRcd**			**pRASRct**		
	***n***	**β (95% CI)**	***p-*value**	**n**	**β (95% CI)**	***p*-value**	**n**	**β (95% CI)**	***p*-value**	**n**	**β (95% CI)**	***p*-value**	**n**	**β (95% CI)**	***p*-value**	**n**	**β (95% CI)**	***p*-value**
Year of publication	14	−0.54 (−1.72, 0.63)	0.364	7	−0.78 (−1.75, 0.19)	0.114	13	0.53 (−0.34, 1.41)	0.233	6	0.01 (−0.04, 0.05)	0.805	6	0.01 (−0.10, 0.12)	0.806	7	0.01 (−0.12, 0.15)	0.844
Number of participants	14	0.00 (−0.01,0.01)	0.539	7	0.00 (−0.01,0.00)	0.134	13	0.01 (0.01,0.01)	<0.001	6	0.00 (0.00,0.00)	0.464	6	0.00 (−0.01,0.01)	0.917	7	0.00 (−0.01,0.01)	0.440
Age	14	0.22 (−0.13, 0.58)	0.221	7	0.08 (−0.36, 0.52)	0.719	13	0.22 (−0.05, 0.50)	0.115	6	0.00 (−0.01, 0.01)	0.920	6	0.01 (−0.01, 0.03)	0.406	7	0.01 (−0.01, 0.03)	0.469
Sex (Male)	14	−0.12 (−0.23, −0.01)	0.028	7	−0.06 (−0.17, 0.05)	0.310	13	−0.06 (−0.14, 0.01)	0.111	6	0.00 (0.00, 0.00)	0.387	6	0.00 (−0.01, 0.00)	0.140	7	0.00 (−0.01, 0.01)	0.950
Heart rate, bpm	12	0.11 (−0.89, 1.1)	0.830	6	−0.45 (−1.08, 0.19)	0.166	11	0.46 (0.13, 0.80)	0.007	6	0.00 (−0.01, 0.02)	0.722	6	0.00 (−0.06, 0.06)	0.989	7	−0.04 (−0.10, 0.01)	0.126
BMI, kg/m^2^	9	−0.92 (−3.73, 1.89)	0.520	4	−0.61 (−1.43, 0.21)	0.146	7	−1.44 (−2.74, −0.14)	0.030	6	0.01 (−0.04, 0.07)	0.592	6	0.01 (−0.12, 0.14)	0.897	6	−0.03 (−0.19, 0.14)	0.760
BSA, m^2^	9	−1.16 (−32.67, 30.35)	0.942	6	19.72 (−1.59, 41.02)	0.070	10	−27.23 (−49.83, −4.64)	0.018	3	0.12 (−0.98, 1.23)	0.826	3	−0.85 (−2.31, 0.60)	0.251	4	1.49 (−1.29, 4.26)	0.295
SBP, mmHg	11	−1.01 (−2.08, 0.07)	0.067	7	−0.11 (−1.08, 0.86)	0.828	10	−0.61 (−1.58, 0.36)	0.220	5	0.00 (−0.05, 0.04)	0.855	5	0.06 (0.05, 0.17)	0.264	6	0.05 (−0.08, 0.18)	0.453
DBP, mmHg	11	−1.31 (−2.54, −0.09)	0.036	7	−0.20 (−1.33, 0.92)	0.725	10	−0.71 (−1.89, 0.48)	0.242	5	0.01 (−0.02, 0.05)	0.416	5	−0.05 (−0.17, 0.06)	0.370	6	−0.05 (−0.16, 0.07)	0.433
LVEF, %	8	0.44 (−1.39, 2.28)	0.636	4	0.87 (0.18, 1.56)	0.014	8	−0.32 (−1.09, 0.44)	0.409	4	−0.02 (−0.07, 0.03)	0.450	4	−0.07 (−0.26, 0.11)	0.419	5	0.05 (−0.17, 0.27)	0.663
RAVI, mL/m^2^	9	−0.21 (−2.14, 1.73)	0.835	6	2.09 (0.50, 3.68)	0.010	9	−1.75 (−3.89, 0.40)	0.111	3	0.04 (−0.01, 0.09)	0.133	3	0.01 (−0.04, 0.07)	0.677	4	−0.03 (−0.19, 0.14)	0.751
RVFAC, %	7	0.54 (−0.26, 1.33)	0.185	3	0.31 (−1.13, 1.75)	0.675	7	−0.40 (−0.70, −0.11)	0.007	3	0.01 (−0.01, 0.03)	0.477	3	0.04 (0.00, 0.08)	0.031	4	0.03 (0.01, 0.05)	0.003
TAPSE, mm	9	−1.60 (−3.78, 0.59)	0.153	4	−0.60 (−4.20, 3.00)	0.742	8	0.26 (−0.75, 1.26)	0.616	4	−0.01 (−0.05, 0.04)	0.784	4	−0.03 (−0.15, 0.08)	0.591	4	−0.07 (−0.07, −0.19)	0.272
RVSm, cm/s	7	1.11 (−7.89, 10.12)	0.808	2	–	–	6	−1.65 (−4.79, 1.48)	0.302	3	−0.13 (−0.33, 0.08)	0.228	3	−0.49 (−1.73, 0.75)	0.442	3	0.34 (−1.14, 1.83)	0.651
SPAP, mmHg	6	−0.13 (−1.47, 1.21)	0.851	3	3.28 (−9.71, 16.26)	0.621	5	0.63 (−2.74, 4.00)	0.713	3	0.03 (−0.02, 0.07)	0.317	3	0.03 (−0.02, 0.08)	0.201	3	−0.08 (−0.21, 0.05)	0.235
Gating	13	2.74 (−5.07, 10.55)	0.492	7	−3.97 (−12.41, 4.46)	0.356	13	2.03 (−3.95, 8.02)	0.505	5	0.06 (−0.18, 0.30)	0.638	5	−0.05 (−0.37, 0.26)	0.748	6	−0.11 (−0.65, 0.43)	0.692
Software	12	−1.59 (−8.60, 5.43)	0.658	6	5.13 (0.44, 9.82)	0.032	12	−4.64 (−10.13, 0.85)	0.097	5	−0.11 (−0.28, 0.06)	0.195	5	−0.31 (−0.64, 0.01)	0.056	6	0.01 (−0.53, 0.54)	0.975
Method of calculation	9	5.50 (−1.64,12.65)	0.131	4	2.10 (0.69, 3.50)	0.004	9	3.89 (1.83, 5.95)	<0.001	5	0.10 (−0.04, 0.23)	0.167	5	0.17 (0.03, 0.31)	0.018	6	−0.36 (−0.94,0.22)	0.220

*BMI, Body mass index; BSA, Body surface area; DBP: Diastolic blood pressure; FR, Frame rate; HR, Heart rate; LVEF, Left ventricular ejection fraction; NR, Not reported; pRASRr, peak Right atrial strain rate during the reservoir phase; pRASRcd, peak Right atrial strain rate during the conduit phase; pRASRct, peak Right atrial strain rate during the contraction phase; RASr, Right atrial strain during the reservoir phase; RAScd, Right atrial strain during the conduit phase; RASct, Right atrial strain during the contraction phase; RAVI, Right atrial volume index; RV, Right ventricle; RVFAC, Right ventricular fractional area change; RVSm, Right ventricular systolic velocity; SBP, Systolic blood pressure; SPAP, Systolic pulmonary artery pressure; TAPSE, Tricuspid annular plane systolic excursion*.

#### Conduit Function Markers

The LV ejection fraction (β = 0.87; *P* = 0.014), the RA volume index (β = 2.09; *P* = 0.010), the software used for analysis (β = 5.13; *P* = 0.032), and the method of global value calculation (β = 2.10; *P* = 0.004) were the sources of inter-study heterogeneity for RAScd. In regard to pRASRcd, the RV fractional area change (β = 0.04; *P* = 0.031) and the method of global value calculation (β = 0.17; *P* = 0.018) were the sources of between-study heterogeneity (Table 2).

#### Contraction Function Markers

With respect to RASct, the number of study participants (β = 0.01; *P* < 0.001), the heart rate (β = 0.46; *P* = 0.007), the body mass index (β = −1.44; *P* = 0.030), the body surface area (β = −27.23; *P* = 0.018), the RV fractional area change (β = −0.40; *P* = 0.007), and the method of global value calculation (β = 3.89; *P* < 0.001) were the sources of between-study heterogeneity. The RV fractional area change (β = 0.03; *P* = 0.003) was the source of heterogeneity for pRASRct (Table 2).

#### Publication Bias

Publication bias was non-significant for RASr (*P* for Egger's test = 0.744), RAScd (*P* for Egger's test = 0.580), RASct (*P* for Egger's test = 0.400), RASRr (*P* for Egger's test = 0.290), RASRcd (*P* for Egger's test = 0.856), and RASRct (*P* for Egger's test = 0.118).

#### Study Quality Assessment

The studies incorporated in the present meta-analysis fulfilled six to nine criteria among the 11 proposed quality criteria. All the studies fulfilled more than 50% of the proposed quality criteria. One study fulfilled nine criteria (82%), five studies fulfilled eight (73%), four studies fulfilled seven (64%), and five studies fulfilled six (55%). All the studies defined their objectives, outcomes, confounders, main findings, and strain imaging protocols (Supplementary Table 2).

## Discussion

To the best of our knowledge, we are the first to present the normal ranges of all RA phasic functions (strain and strain rate) through a meta-analysis. A cardiac cycle includes interactions between the RA and the RV. In the RV systolic time, the tricuspid annulus is pulled toward the cardiac apex concurrently with the entrance of the flow from the cava veins into the RA. In this phase (the reservoir phase), the RA myocardium is stretched. In the early RV diastolic time, which is concurrent with the RA conduit phase, the tricuspid valve opens and the tricuspid annulus returns to its original place due to RV relaxation (reduced RA stretching). In the late RV diastolic time, which coincides with the RA contraction phase, the RA contracts, and the length of the RA myocardial fibers decreases (1).

The markers of the 3 RA phasic functions are predictive of prognosis in patients with pulmonary hypertension, while the markers of the RA reservoir and conduit functions are correlated with functional capacity in patients with systemic sclerosis and idiopathic pulmonary hypertension (25, 31–34). In addition, RASr correlates with the RA pressure and the occurrence of postoperative atrial fibrillation (35, 36).

### Sources of Heterogeneity

#### The Reservoir Function

According to some studies, RASr increases in women in comparison with men (4, 11). We found that sex was a source of inter-study variation (37).

In a previous investigation, RASr was decreased in patients with high normal blood pressure, defined as a diastolic blood pressure of about 9 mm Hg higher than the normal diastolic blood pressure in the control group (79 vs. 70 mm Hg), during a 24-h blood pressure monitoring (38). Our findings are in line with that study insofar as the range of the diastolic blood pressure in our study was between 71 and 81 mm Hg.

#### The Conduit Function

The RA conduit function is correlated with the LV ejection fraction in patients with a reduced ejection fraction (2). In our meta-analysis, we assessed studies that enrolled patients with normal cardiac function. Still, the LV ejection fraction in these studies ranged from 55 to 65%.

A previous investigation demonstrated that during the head-up title test with increased inclination, the LA volume and conduit function decreased (7). Stated otherwise, a decrease in the LA volume is in tandem with a reduction in the LA conduit function. Concerning the RA, in patients with end-stage renal disease, the RA volume and conduit function decrease after hemodialysis, which is compatible with the aforementioned study regarding the LA (39). It can, therefore, be explained why the inter-study difference vis-a-vis the RA volume index may lead to an inter-study difference in terms of the RA conduit function.

The LV systolic function is a determinant of the LA conduit function (40), and the RV fractional area change is a marker of the RV systolic function (41). Hence, it is reasonable that the RV systolic function is a determinant of the RA conduit function and that the difference regarding the RV fractional area change between studies is a source of between-study heterogeneity.

The global strain can be calculated by two methods, one of which uses the entire myocardial line length while computing the global strain, whereas the other one averages the values computed at the segmental level. These methods are mathematically the same. Nevertheless, in practice, the presence of a bad track or noisy signal segment can be a source of difference between these two methods. This segment is included in the calculation in the first method and excluded in the second method. More differences manifest themselves when the segments are not equal in size and the weight of small and large segments is similar (42). Indeed, such differences in the methodology of global strain calculation can be a source of between-study heterogeneity.

The software used by researchers was another source of heterogeneity. Intervendor variability is mainly due to post-processing factors such as differences in algorithms and the calculation methods of deformation markers, as well as the control of outliers and the order in which deformation markers are calculated (43).

#### The Contraction Function

Some studies have reported that a rise in the body mass index is accompanied by a decline in the LA contraction function (44, 45), which can be used as a rationale to assume that a decrease in the RA contraction function is allied to an increase in the body mass index. This may explain why the body mass index was a source of inter-study heterogeneity in our meta-analysis. The correlation between the body mass index and the body surface area may render the latter a source of between-study variabilities. In addition, with an increase in body size, the initial length of the RA myocardium increases, leading to a decrease in strain since strain is the ratio of length change to the initial length.

The number of participants can be deemed a measure of researchers' expertise in the measurement of strain and strain rates (14). The level of expertise may, thus, affect the results of studies. Studies do not tend to mention the level of their researchers' expertise, and a consensus has yet to emerge regarding the objective criteria of expertise in this field.

Whereas some studies claim that there is a correlation between the heart rate and aggravation in atrial contraction, some other studies refute this notion (27, 46). According to our results, an increase in age was correlated with an increase in the RA contraction function; be that as it may, this association cannot be considered etiologic because what we sought to determine was the source of heterogeneity between studies. The range of the reported heart rate in our study was between 63 and 76 beats per minute, which is acceptable for the normal population.

As was mentioned above, based on our findings, the RV fractional area change was a source of between-study heterogeneity inasmuch as an increase in the RV fractional area change was correlated with an increase in the RA conduit function. While the RV fractional area change was a source of heterogeneity concerning the RA conduit function, an increased RV fractional area change was associated with a decrease in the RA contraction function. This finding can be explained by the fact that with an increase in the conduit phase for RV filling, the RA contraction function decreases and vice versa. This issue is seen in the case of grade I LV diastolic dysfunction, in which a decrease in LV diastolic filling in the early diastole is in tandem with an increase in LV diastolic filling in the late diastole due to the contraction of the LA.

#### Publication Bias

We found no publication bias. However, the low number of studies, especially in the case of the RA phasic strain rate, undermines the accuracy of our results.

Our study selection was done independently. All the stages of study selection were checked by three researchers, and any discrepancy was ultimately removed through an agreement between two researchers. We think that this adopted method minimized the possibility of missing available studies.

Our study yielded normal ranges for the longitudinal markers of the RA phasic functions obtained by 2DSTE. Although we analyzed a limited number of studies, our study proved preliminary data regarding the normal ranges of these markers. Further research is needed to obtain more robust data on the normal ranges of these markers in different populations. Our study can be helpful for clinicians interested in right heart disease in that it defines what constitutes normal ranges for RA phasic functions. Additionally, information on the normal ranges of the deformation markers of the RA phasic functions may be helpful in patient follow-up since it could identify the time of RA involvement in the disease process. Another salient point raised by our study is the need for further well-designed studies in this field.

### Study Limitations

Fifteen studies that included 2,469 healthy subjects were selected for our meta-analysis concerning various markers of the RA phasic functions (strain and strain rate). Despite the low sample size of our investigation, we hope that until larger meta-analyses are undertaken, clinicians will find our results useful. In contrast with previous meta-analyses that included randomized clinical trials, our meta-analysis evaluated observational and case-control studies. Accordingly, the fact that we encountered a high rate of heterogeneity by comparison with the meta-analyses that evaluated randomized clinical trials can be deemed expectable (47).

The expertise level of researchers is another matter that is obscure in studies in this field and should be considered in future studies. The majority of the studies included in the meta-analysis had small patient populations, which further underscores the need for large-scale investigations in this context. What should also be borne in mind in the interpretation of our findings is that we analyzed merely the data of studies and not the data of patients who participated in the studies. Moreover, the quality of the studies subjected to analysis was evaluated through checklists (14, 48). Such checklists offer an insight into the quality of studies, but they lack objectivity in some items (49). Notably, we did not consider the quality of a study an exclusion criterion. The overall quality of the studies meta-analyzed herein was acceptable because they fulfilled six to nine criteria among the 11 proposed quality criteria. In addition, we did not consider the quality of study in our analysis and did not a stratified meta-analysis.

## Conclusions

According to the results of the present study, the mean global value was 42.7% for RASr (95% CI, 39.4 to 45.9%), 23.6% for RAScd (95% CI, 20.7 to 26.6%), 16.1% for RASct (95% CI, 13.6 to 18.6%), 2.1 s^−1^ for pRASRr (95% CI, 2.0 to 2.1 s^−1^), −1.9 s^−1^ for pRASRcd (95% CI, −2.2 to −1.7 s^−1^), and −1.8 s^−1^ for pRASRct (95% CI, −2.0 to −1.5 s^−1^). The sources of heterogeneity in terms of the normal ranges of these markers were the number of participants, the type of software, the method of global value calculation, the RV fractional area, the LV ejection fraction, the RA volume index, sex, the heart rate, the diastolic blood pressure, the body mass index, and the body surface area.

## Author Contributions

AH, TD, and RM-B: concept/design. AH, TD, RM-B, RM, and AJ: data analysis/interpretation and approval of article. AH: drafting article. TD, RM-B, RM, and AJ: critical revision of article. AJ: statistics. AH, RM, and RM-B: data collection. All authors contributed to the article and approved the submitted version.

## Conflict of Interest

The authors declare that the research was conducted in the absence of any commercial or financial relationships that could be construed as a potential conflict of interest.

## Publisher's Note

All claims expressed in this article are solely those of the authors and do not necessarily represent those of their affiliated organizations, or those of the publisher, the editors and the reviewers. Any product that may be evaluated in this article, or claim that may be made by its manufacturer, is not guaranteed or endorsed by the publisher.
