# Supplementary material for: Normal Ranges of Right Atrial Strain and Strain Rate by Two-Dimensional Speckle-Tracking Echocardiography: A Systematic Review and Meta-Analysis
Source: Front Cardiovasc Med. 2021 Dec 17;8:771647. doi: 10.3389/fcvm.2021.771647 (PMC8718502; doi:10.3389/fcvm.2021.771647)
Supplement: Supplementary file 1 [file Table_1.docx]

**Table 1S**: Full text exclusion reasons

| First Author | Year of Publication | Exclusion Reasons |
| --- | --- | --- |
| D’Andrea | 2009 | No global strain results, only base of lateral wall was considered |
| Riesenkampff | 2010 | Some subjects were not in adult range (age =24.6 ± 8.7) |
| Ojaghi Haghighi | 2011 | No 2DSTE data |
| Liang | 2011 | No 2DSTE data |
| Zheng | 2011 | No 2DSTE data |
| Yan | 2012 | No healthy control |
| Goebel | 2013 | Hypertension and diabetes were not excluded |
| Ren | 2014 | No 2DSTE data |
| Tadic | 2014 | No healthy control |
| Tadic | 2014 | No healthy control |
| Tadic | 2014 | Repeated data set |
| Tadic | 2014 | Repeated data set |
| Tadic | 2014 | Repeated data set |
| Tadic | 2014 | Repeated data set |
| Abd El Rahman | 2015 | Control group age < 18 y |
| Bech-Hanssen | 2015 | Only measure two segments in medal and lateral wall |
| Moustafa | 2015 | Discrepancy between text and figures regarding measurement method |
| Nemes | 2015 | No 2DSTE data |
| Tadic | 2015 | Repeated data set |
| Tigen | 2015 | Hypertension and diabetes were not excluded |
| Addetia | 2016 | Hypertension and diabetes were not excluded |
| D’Ascenzi | 2016 | Some control case seems to be < 18 years |
| D’Andrea | 2016 | Repeated data set |
| Kado | 2016 | Repeated data set |
| Muraru | 2016 | RA strain results were not presented |
| Ozkan | 2016 | No healthy control |
| Sanz-de la Garza | 2016 | Participants were athletes |
| Sakata | 2016 | Hypertension and diabetes were not excluded |
| Tadic | 2016 | Repeated data set |
| Tadic | 2016 | Repeated data set |
| To | 2016 | Hypertension and diabetes were not excluded |
| Wang | 2016 | No healthy control |
| Amsallem | 2017 | No 2DSTE data |
| Amenabar | 2017 | Hypertension and diabetes were not excluded |
| Bhave | 2017 | Hypertension and diabetes were not excluded |
| Hernandez-Suarez | 2017 | No 2DSTE data |
| Kutty | 2017 | No 2DSTE data |
| Piccinino | 2017 | Hypertension and diabetes were not excluded |
| Ramberg | 2017 | No healthy control |
| Sanchis | 2017 | Data of male and female presented separately |
| Sareban | 2017 | Hypertension and diabetes were not excluded |
| Brand | 2018 | No healthy control |
| Deschle | 2018 | Hypertension and diabetes were not excluded |
| Gunay | 2018 | No 2DSTE data |
| Hubert | 2018 | No healthy control |
| Khan | 2018 | Hypertension and diabetes were not excluded |
| Tadic | 2018 | Repeated data set |
| Weiwei | 2018 | Hypertension and diabetes were not excluded |
| Meng | 2018 | Hypertension and diabetes were not excluded |
| Nogradi | 2018 | Hypertension and diabetes were not excluded |
| Sun | 2018 | Hypertension and diabetes were not excluded |
| Vitarelli | 2018 | Hypertension and diabetes were not excluded |
| Wright | 2018 | No healthy control |
| Akazawa | 2019 | No 2DSTE data |
| Cheung | 2019 | Hypertension and diabetes were not excluded |
| Dąbrowska-Kugacka | 2019 | Some subjects were not in adult range (age =19.7 ± 8.5) |
| Dordevic | 2019 | Hypertension were not excluded |
| Gurbuz | 2019 | No 2DSTE data |
| Hajizeinali | 2019 | Only RA lateral wall deformation markers were presented |
| Kishiki | 2019 | Hypertension and diabetes were not excluded |
| Safir-Mardanloo | 2019 | No 2DSTE data |
| Zhang | 2019 | Hypertension and diabetes were not excluded |
| Cincin | 2020 | No healthy control |
| Huang | 2020 | Diabetes were not excluded |
| Li | 2020 | Some subjects were not in adult range (age =26.1 ± 9.8) |
| Marques‑Alves | 2020 | No healthy control |
| Sonaglioni | 2020 | No healthy control |
| Sareban | 2020 | Hypertension and diabetes were not excluded |
| Theres | 2020 | No healthy control |
| Truong | 2020 | Repeated data set |
| Goedemans | 2021 | Some control subjects suffer thyroid disease |
| He | 2021 | Hypertension and diabetes were not excluded |
| He | 2021 | Hypertension and diabetes were not excluded |
| Hasselberg | 2021 | Hypertension and diabetes were not excluded |
