# Supplementary material for: Normal Ranges of Right Atrial Strain and Strain Rate by Two-Dimensional Speckle-Tracking Echocardiography: A Systematic Review and Meta-Analysis
Source: Front Cardiovasc Med. 2021 Dec 17;8:771647. doi: 10.3389/fcvm.2021.771647 (PMC8718502; doi:10.3389/fcvm.2021.771647)
Supplement: Supplementary file 2 [file Table_2.docx]

**Table 2S**: Quality evaluation of included article

| Study | Year | Total number of “Yes” in evaluation by quality check list | Objective  defined | Outcome  described | Characteristics  of controls  described | Confounders  described | Main  findings  outlined | Heterogeneous  population | Strain  imaging  protocol | Individuals  generating data  blinded to  outcomes | Sonographers  blinded to  outcome | Was  reproducibility  analysis  performed? | Patients/control  subjects  recruited over  same time  period |
| --- | --- | --- | --- | --- | --- | --- | --- | --- | --- | --- | --- | --- | --- |
| Padeletti et al | 2012 | 8 | Yes | Yes | Yes | Yes | Yes | NS | Yes | No | No | Yes | Yes |
| D’Ascenzi et al | 2013 | 6 | Yes | Yes | No | Yes | Yes | NS | Yes | Yes | No | No | No |
| Peluso et al | 2013 | 8 | Yes | Yes | Yes | Yes | Yes | NS | Yes | No | No | Yes | Yes |
| Pagourelias et al | 2013 | 7 | Yes | Yes | Yes | Yes | Yes | NS | Yes | No | No | Yes | No |
| Gabrielli et al | 2014 | 7 | Yes | Yes | No | Yes | Yes | NS | Yes | Yes | No | Yes | No |
| Durmus et al | 2015 | 6 | Yes | Yes | No | Yes | Yes | NS | Yes | No | No | Yes | No |
| McClean et al | 2015 | 6 | Yes | Yes | Yes | Yes | Yes | NS | Yes | No | No | No | No |
| Querejeta Roca et al | 2015 | 8 | Yes | Yes | Yes | Yes | Yes | NS | Yes | Yes | No | Yes | No |
| Tadic et al | 2015 | 7 | Yes | Yes | Yes | Yes | Yes | NS | Yes | No | No | Yes | No |
| Gabrielli et al | 2016 | 8 | Yes | Yes | Yes | Yes | Yes | NS | Yes | No | Yes | Yes | No |
| Brand et al | 2018 | 8 | Yes | Yes | Yes | Yes | Yes | NS | Yes | No | No | Yes | Yes |
| Li et al | 2018 | 6 | Yes | Yes | Yes | Yes | Yes | NS | Yes | No | No | No | No |
| Can Bostan et al | 2020 | 7 | Yes | Yes | Yes | Yes | Yes | NS | Yes | No | No | Yes | No |
| Palmer et al | 2020 | 6 | Yes | Yes | Yes | Yes | Yes | NS | Yes | No | No | No | No |
| Soulat-Dufour et al | 2020 | 9 | Yes | Yes | Yes | Yes | Yes | NS | Yes | Yes | Yes | No | Yes |
