# Supplementary material for: Normal Ranges of Right Atrial Strain and Strain Rate by Two-Dimensional Speckle-Tracking Echocardiography: A Systematic Review and Meta-Analysis
Source: Front Cardiovasc Med. 2021 Dec 17;8:771647. doi: 10.3389/fcvm.2021.771647 (PMC8718502; doi:10.3389/fcvm.2021.771647)
Supplement: Supplementary file 3 [file Data_Sheet_1.docx]

**Supplement**:

**PubMed**

("Right atrial" OR "right atrium") AND (strain or speckle or deformation) AND echocardiography

Filter: the English Language

PubMed: 1801

**Scopus**

(TITLE-ABS-KEY ("Right atrial" OR "right atrium")) AND (TITLE-ABS-KEY (strain OR speckle OR deformation)) AND (TITLE-ABS-KEY (“echocardiography”))

Filter: the English Language

Scopus: 418

**Embase**

Quick search:

("Right atrial" OR "right atrium") AND (strain or speckle or deformation) AND echocardiography

Filter: the English Language

Embase: 971
